# From respiratory viral infection to persistent cough hypersensitivity: neuroimmune regulation and therapeutic implications of P2X3/TRP ion-channel targets

**DOI:** 10.3389/fphar.2026.1873086

**Published:** 2026-09-29

**Authors:** Borui Li, Ziyang Yu, Fanshuai Zeng, Xiaotian Jiang, Haotian Xi, Kun Wang, Lixiu Zhang

**Affiliations:** 1 Changchun University of Chinese Medicine, Changchun, China; 2 Jilin Provincial Center for Drug Review and Inspection, Changchun, China; 3 Affiliated Hospital of Changchun University of Chinese Medicine, Changchun, China

**Keywords:** cough hypersensitivity syndrome, P2X3 receptor, P2X3/TRP, post-infectious cough, TRP channels

## Abstract

Respiratory viral infections are increasingly recognized as important drivers of persistent airway symptoms and exacerbations in chronic lung disease. Post-infectious cough (PIC), particularly after upper respiratory tract infection, represents one of the most frequent causes of subacute cough and a mechanistically complex sequela in which cough hypersensitivity syndrome (CHS) plays a central pathophysiological role. Beyond increased cough frequency, patients with PIC frequently exhibit laryngeal sensory abnormalities and exaggerated responses to low-intensity stimuli, suggesting a sustained reduction in the activation threshold of airway sensory nerves. Among several contributors to cough hypersensitivity, P2X3 receptors and transient receptor potential (TRP) channels are key ion-channel targets mediating cough reflexes and sensory nerve hypersensitivity, yet their clinical translation has been heterogeneous. Gefapixant reduced objective cough frequency in phase III trials enrolling refractory or unexplained chronic cough populations, but these data provide indirect rather than PIC-specific evidence, and its clinical utility is limited by dysgeusia. Similarly, TRPV1 antagonists suppress capsaicin-provoked cough but have not consistently improved spontaneous cough frequency, highlighting a disconnect between provocation models and real-world post-infectious cough pathology. This review discusses how respiratory viruses may induce cough hypersensitivity through transcriptional, metabolic, and neuroimmune regulation of sensory pathways. Respiratory syncytial virus, rhinovirus, and other viral pathogens can upregulate TRPV1, TRPA1, and ASIC3 expression, while cytokines such as IFN-γ may promote sensory-neuron transcriptional reprogramming through JAK-STAT and NF-κB signaling. In parallel, inflammatory mediators including bradykinin, COX/12-LOX metabolites, and prostaglandin E2 can sensitize TRP channels through kinase-dependent phosphorylation, whereas virus-induced epithelial ATP release and impaired CD39/CD73-mediated purinergic degradation may sustain repeated P2X3 receptor activation. Persistent peripheral afferent input may further induce central sensitization, explaining residual cough despite peripheral target blockade. By integrating viral immunopathogenesis, epithelial–neural crosstalk, and ion-channel pharmacology, this review proposes a framework for understanding post-viral cough hypersensitivity as a neuroimmune sequela of respiratory infection. Future research should prioritize molecular endotype-based stratification, PIC-specific randomized controlled trials, optimization of highly selective P2X3 antagonists, and combined peripheral–central assessment strategies to guide more precise therapeutic interventions.

## 1 Introduction

Understanding of the pathophysiology of chronic cough has expanded in recent years from simple airway inflammation or structural disease to abnormal sensory nerve excitability. Chronic cough is defined by current guidelines as cough lasting 8 weeks or more in adults (Morice et al., 2020; Irwin et al., 2018). Cough hypersensitivity syndrome is used to describe the state in which patients produce abnormal cough responses to low-intensity chemical, mechanical, or thermal stimuli, with its mechanisms involving peripheral sensory nerve sensitization, central cough regulatory abnormalities, and neuroimmune interactions (Morice et al., 2020; Chung et al., 2022; Song and Morice, 2017). The lack of biomarkers available for diagnosis, efficacy prediction, and treatment response monitoring in patients with refractory or unexplained chronic cough remains a key limitation of current clinical management (Hassan et al., 2025). Refractory chronic cough (RCC) refers to cough that persists despite guideline-based treatment of identifiable causes, whereas unexplained chronic cough (UCC) refers to chronic cough without an identifiable cause after appropriate evaluation (Morice et al., 2020). PIC is generally defined as cough lasting 3–8 weeks following the acute symptoms of a respiratory infection, corresponding to the guideline-defined subacute cough spectrum (Irwin et al., 2018; Braman, 2006). When such cough persists beyond 8 weeks, it enters the chronic cough spectrum and may then be classified as UCC if no cause is identified, or as RCC if it persists despite guideline-based treatment (Morice et al., 2020).

Post-infectious cough is an important clinical phenotype of cough hypersensitivity syndrome, typically referring to cough that persists after resolution of acute symptoms of respiratory tract infection. Viral infection may induce cough hypersensitivity through epithelial damage, inflammatory mediator release, and sensory nerve sensitization (Li and Deng, 2022). Epidemiological data indicate that post-infectious cough develops in approximately 11%–25% of adults following acute respiratory infection (Liang et al., 2024) and represents one of the most common causes of subacute cough (Irwin et al., 2018). Accordingly, viral respiratory infection should be viewed not only as the initiating event of PIC but also as an upstream driver of persistent cough that, in susceptible individuals, extends into the chronic cough spectrum (Liang et al., 2024; He et al., 2026). In a recent nationwide prospective cohort of patients with post-COVID-19 persistent cough, cough hypersensitivity to capsaicin and ATP was observed in 72.5% and 63.2% of tested patients, respectively (He et al., 2026), supporting a link between viral infection and the sensory nerve hyperresponsiveness that characterizes chronic cough. Because PIC overlaps clinically with other causes of subacute and chronic cough, careful phenotyping is required. This review therefore focuses on neuroimmune and ion-channel mechanisms that may help distinguish infection-associated cough hypersensitivity from other cough syndromes.

Among the peripheral molecular mechanisms of cough hypersensitivity, the P2X3 receptor and TRP channel family have received extensive attention. The P2X3 receptor primarily senses extracellular ATP released following epithelial damage or stress and participates in the cough reflex through vagal sensory nerve afferents. TRP channels including TRPV1, TRPA1, and TRPV4 respond to multiple factors including capsaicin, acidic environments, high temperatures, oxidative stress products, cold stimuli, osmotic changes, and mechanical stimuli (Xu et al., 2024; Mazzone and Undem, 2016; Bonvini et al., 2016). Both have a robust physiological basis and preclinical evidence, but clinical translation outcomes differ. The P2×3 receptor antagonist gefapixant reduced objective cough frequency in chronic cough patients in phase III clinical trials (McGarvey et al., 2022); whereas TRPV1 antagonists, although capable of suppressing capsaicin-provoked cough, failed to reduce spontaneous cough frequency (Belvisi et al., 2017; Khalid et al., 2014). These differences indicate that single-provocation models cannot fully represent the multifactorial pathological process of real-world chronic cough.

Based on this background, this review addresses a key question: why the hyperexcitability of P2X3 and TRP channels in PIC can persist, and how this sustained hypersensitivity explains the efficacy differences between the two classes of antagonists. The analysis is organized around two dimensions—transcriptional regulation (how viral infection and cytokines alter channel gene expression) and metabolic regulation (how inflammatory mediators and ATP metabolic imbalance lower channel activation thresholds) — and extends to second-generation P2X3 antagonist development, molecular endotype-based stratification, and PIC-specific clinical study design. We distinguish established clinical evidence from indirect RCC/UCC evidence, preclinical model-based evidence, and exploratory mechanisms to avoid overstating findings that have not yet been validated in PIC-specific human cohorts. As this is a narrative review rather than a systematic review or meta-analysis, cross-study comparisons are descriptive and should not be interpreted as quantitative efficacy rankings.

## 2 Clinical manifestations of PIC extend beyond increased cough frequency

### 2.1 Laryngeal sensory abnormalities and low-intensity stimulus-induced cough indicate reduced airway sensory nerve thresholds

The clinical manifestations of PIC and chronic cough patients are frequently not limited to increased cough frequency. Many patients simultaneously present with laryngeal sensory abnormalities, including throat itching, foreign body sensation, burning sensation, dryness, throat-clearing urge, and speech-induced discomfort. A cross-sectional study of hospital workers demonstrated that laryngeal sensory abnormalities were significantly associated with cough occurrence and correlated with factors including asthma, gastroesophageal reflux, and emotional fluctuations (Amakusa et al., 2025). This indicates that cough hypersensitivity may involve multiple levels including upper airway sensory afferents, reflux-related stimuli, and central regulation, rather than being confined to the lower airways.

Beyond spontaneous laryngeal discomfort, patients commonly report cough triggered by cold air, speaking, laughing, odors, smoke, eating, or exercise. This phenomenon of cough induced by low-intensity stimuli indicates a reduction in airway sensory nerve excitation thresholds. A potential link exists between environmental factors and ion channel expression. A recent study demonstrated that dry heat environments can increase the risk of airway hyperresponsiveness and alter expression of genes related to airway sensation or epithelial homeostasis, including TRPV4, PIEZO1, SCNN1A, SCNN1B, ANO1, and CFTR, in airway epithelial cells (Edwards et al., 2025). Although this study was not PIC-specific, it indicates that external physical environments can influence cough susceptibility through epithelial stress and channel expression alterations. Figure 1 provides an overview of the distribution of P2X3 pathways and TRP channel family members at the airway epithelial-sensory nerve interface and their primary stimulus types, facilitating understanding of the correspondence between different trigger factors and different ion channels.

**FIGURE 1:**
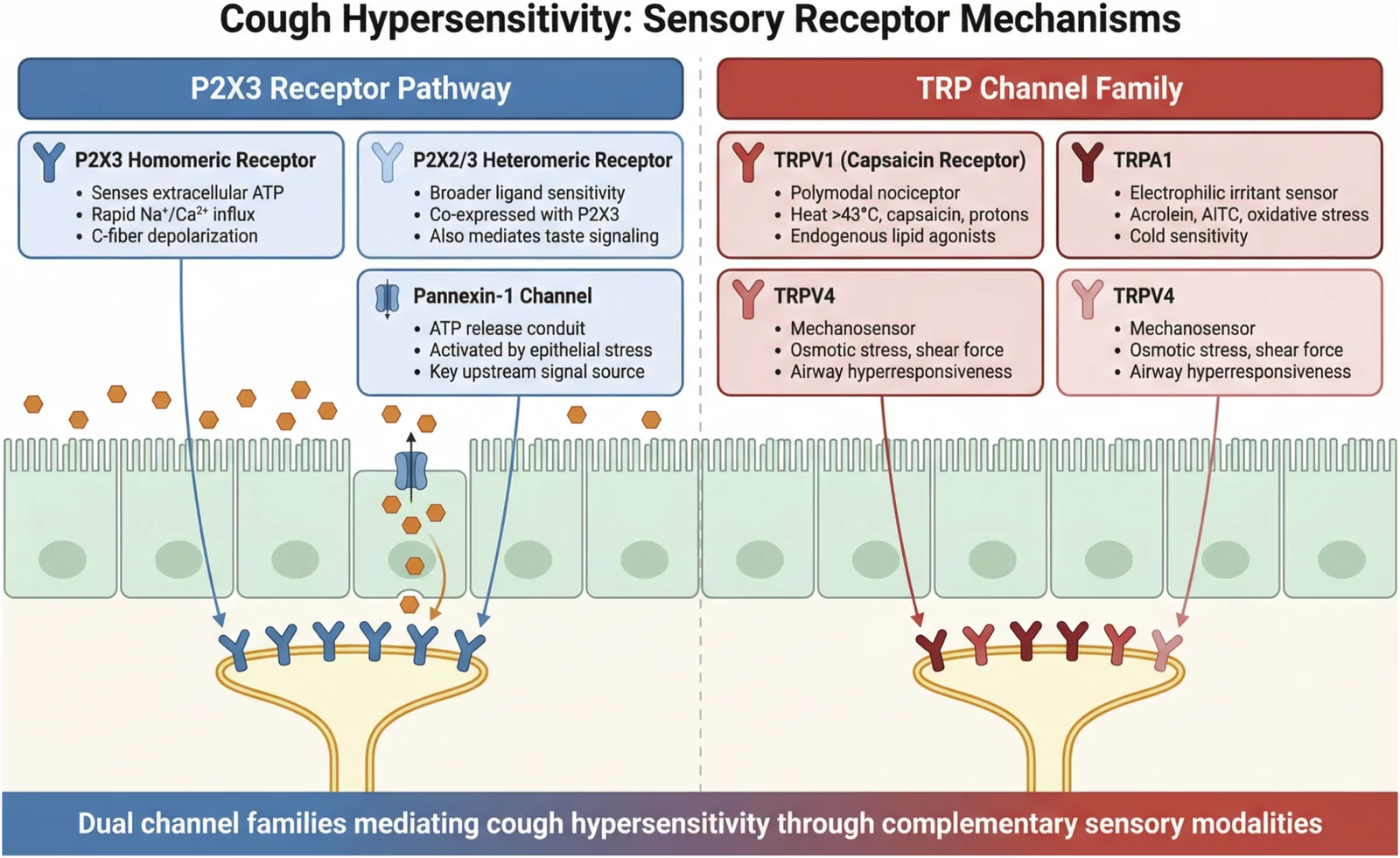
Overview of P2X3 receptor pathways and TRP channel subtypes in airway sensory nerves.

### 2.2 Metabolic background and channel response profiles suggest candidate stratification axes

Treatment responses in chronic cough and PIC exhibit substantial interindividual variability. Even when patients are classified within the same clinical diagnostic category, their responses to anti-inflammatory therapy, neuromodulatory agents, P2X3 antagonists, or stimulus avoidance strategies may differ. This therapeutic heterogeneity suggests that a single clinical diagnostic label may encompass multiple molecular endotypes driven by different pathways. Metabolic background may act as a risk modifier for heterogeneity in PIC. In an obese population, increased waist circumference and insulin resistance were associated with a higher risk of PIC after upper respiratory tract infection (Xian et al., 2025). A plausible link is that insulin resistance is accompanied by low-grade systemic inflammation, oxidative stress, and epithelial barrier dysfunction, which may lower the threshold for airway sensory nerve activation. However, direct evidence that insulin resistance alters P2X3 or TRP-channel expression in human PIC remains lacking.

Channel response profile differences also support the necessity of molecular subtyping. Cough sensitivity to the TRPV1 agonist capsaicin and the TRPA1 agonist AITC is not entirely consistent among patients with chronic refractory cough; some patients are highly sensitive to capsaicin but show low responses to AITC, while others display the opposite pattern (Long et al., 2019). This indicates that TRPV1- and TRPA1-mediated cough hypersensitivity may represent different endotypes. These findings indicate that biomarkers capable of accurately assigning patients to corresponding pathway-targeted therapies are currently lacking. Thus, metabolic status and channel-provocation profiles should be viewed as candidate stratification axes rather than validated molecular endotypes.

## 3 P2X3 signaling preferentially mediates C-fiber afferents; ATP is the key mediator of epithelial-neural communication

### 3.1 C-fibers mediate chemosensory perception; P2X3 signaling preferentially transmits endogenous damage signals

Airway cough afferents comprise unmyelinated C-fibers, which express TRPV1 and TRPA1 and respond to chemical and inflammatory stimuli, and thinly myelinated Aδ-fibers, which are primarily mechanosensory (Xu et al., 2024).

P2X3 receptors are expressed across multiple neuronal types and participate in neuronal excitability regulation (Bussey et al., 2026). Within the airway afferent nervous system, ATP-P2X3 signaling is of considerable importance for bronchopulmonary C-fiber excitation. Studies in human and guinea pig airways demonstrated that mechanical stimulation and hypotonic stimulation can promote ATP release from airway epithelium, and P2X receptor antagonists can suppress ATP analog-evoked action potential discharge in bronchopulmonary nodose ganglion C-fibers, but have limited effects on the excitability of certain extrapulmonary Aδ-fibers (Canning et al., 2026). These results indicate that P2X3 signaling may preferentially regulate C-fiber afferents driven by endogenous damage or stress signals, rather than fully covering all protective cough pathways. Figure 1 illustrates the functional division of labor among P2X3, P2X2/3, TRPV1, TRPA1, and TRPV4 within the airway epithelial-sensory nerve unit.

### 3.2 Epithelial ATP release activates sensory nerve terminals via P2X3 receptors; central sensitization may contribute to residual cough

ATP is an important mediator connecting epithelial damage with sensory nerve excitation. Under physiological conditions, extracellular ATP concentrations are low; when airway epithelium is subjected to viral infection, mechanical stretch, osmotic changes, inflammatory mediators, or allergen stimulation, ATP can be released into the extracellular environment through Pannexin channels and other pathways (Canning et al., 2026). ATP subsequently acts on P2X3 receptors at sensory nerve terminals, triggering cation influx, membrane depolarization, and action potential discharge; afferent signals enter brainstem cough-related neural circuits via the vagus nerve, ultimately participating in cough reflex formation.

Epithelium-derived ATP release has been supported by substantial experimental evidence. Removal of airway epithelium substantially attenuates ATP release induced by mechanical stimulation and hypotonic stimulation, indicating that the epithelium is an important source of this signal (Canning et al., 2026). The effects of increased ATP release, decreased degradation, and sustained P2X3 activation under pathological conditions are discussed in Section 7.2.

Beyond peripheral afferent enhancement, central sensitization may also contribute to the maintenance of cough hypersensitivity. Sustained peripheral stimulation can enhance input to brainstem and higher cortical cough regulatory networks, inducing synaptic plasticity changes that elevate central gain of the cough reflex (Chung et al., 2022; Mazzone and Undem, 2016). Functional brain imaging studies have demonstrated that chronic cough patients may exhibit functional abnormalities in brain regions related to cough urge and inhibitory control (Mazzone et al., 2007; Ando et al., 2016). This indicates that even when peripheral targets are suppressed, some patients may continue to experience residual cough or cough urge. Therefore, a more complete mechanistic model of PIC and chronic cough should consider both peripheral sensory nerve sensitization and central cough regulatory alterations.

## 4 P2×3 antagonists reduce objective cough frequency, but dysgeusia limits broader application

### 4.1 Dose-dependent reduction in cough frequency by gefapixant

The P2X3 receptor antagonist gefapixant has provided pivotal clinical evidence for neural-targeted therapy of chronic cough. COUGH-1 and COUGH-2 are two global multicenter, randomized, double-blind, placebo-controlled phase III clinical trials that enrolled adult patients with refractory or unexplained chronic cough, with a mean cough history of approximately 11 years and a predominantly female population (McGarvey et al., 2022; Muccino et al., 2020). Both studies used objectively monitored 24-h cough frequency as the primary efficacy endpoint.

Results demonstrated that gefapixant 45 mg twice daily significantly reduced 24-h cough frequency relative to placebo at week 12 in COUGH-1 and week 24 in COUGH-2, with relative reductions of 18.5% and 14.6%, respectively (McGarvey et al., 2022). The 15 mg twice daily dose did not meet the primary endpoint in both studies. These results support the ATP-P2X3 axis as a targetable pathway in refractory or unexplained chronic cough. Figure 2 outlines the clinical translation differences between P2X3 antagonists and TRPV1 antagonists.

**FIGURE 2:**
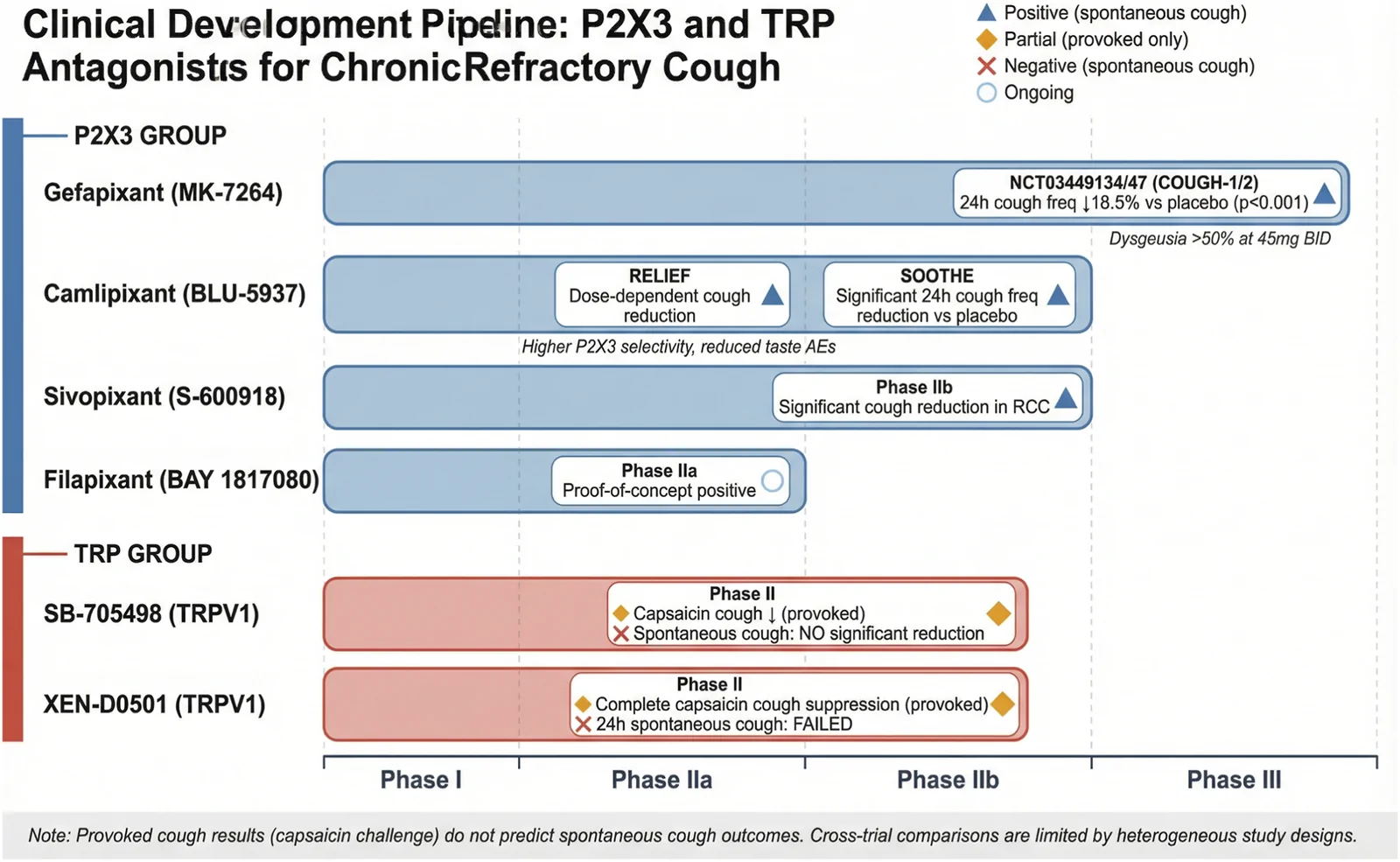
Summary of clinical trial evidence for P2X3 antagonists and TRPV1 antagonists in chronic cough. Symbols indicate positive provocation-test effects and negative spontaneous-cough outcomes, respectively.

### 4.2 P2X2/3 off-target inhibition causes dysgeusia; taste bud and antitussive pathways require separation

The primary limitation of gefapixant is taste-related adverse events. In COUGH-1 and COUGH-2, dysgeusia, hypogeusia, ageusia, and taste distortion were common in the 45 mg twice daily group, with an overall incidence exceeding 50% (McGarvey et al., 2022). A Japanese 52-week long-term safety study also demonstrated that taste-related adverse event incidence reached 96% in the 45 mg group, with approximately 20% of patients discontinuing treatment for this reason (Niimi et al., 2022). Although most taste-related events were mild to moderate, their high incidence adversely affects long-term adherence and patient acceptability.

The pharmacological basis of dysgeusia is thought to be related, at least in part, to inhibition of P2X2/3 heterotrimeric receptors involved in taste-bud signaling (Finger et al., 2005). Insufficient discrimination between P2X3 homotrimers and P2X2/3 heterotrimers may therefore suppress ATP-mediated taste transmission while also inhibiting cough-relevant vagal afferent pathways (Friedri et al., 2022). The inherent validity of the P2X3 target must be distinguished from the off-target inhibition of P2X2/3 by first-generation agents. Section 8.2 discusses how highly selective P2X3 antagonists may preserve antitussive effects while reducing taste-related adverse events.

## 5 TRPV1 antagonists suppress provoked cough but fail to reduce spontaneous cough

### 5.1 TRPV1 and TRPA1 effectively suppress cough in PIC animal models but have not translated to clinical spontaneous cough improvement

The TRP channel family is an important molecular basis for airway sensory nerve perception of external physical and chemical stimuli. TRPV1 can be activated by capsaicin, high temperatures, acidic environments, and multiple endogenous inflammatory mediators, and is therefore also known as the capsaicin receptor (Xu et al., 2024). TRPA1 can sense exogenous irritants including smoke, formaldehyde, and acrolein, and can also be activated by oxidative stress products. TRPV4 is associated with mechanical stimulation, osmotic changes, and epithelial stress (Bonvini et al., 2016; Edwards et al., 2025). These channels have different stimulus profiles in airway C-fibers and epithelial-neural units, which can explain patient sensitivity to odors, cold air, hot air, irritant chemicals, and environmental changes.

PIC animal models and traditional Chinese medicine formula mechanism studies have provided preclinical evidence for TRPV1/TRPA1 involvement in cough hypersensitivity. A study of Kemin Capsule demonstrated that modulating PI3K/AKT signaling and downregulating TRPV1/TRPA1-related expression improved cough and inflammatory responses in PIC models (Yang et al., 2025). A study of Zhisou Powder also indicated that suppressing TRPA1/TRPV1 channels and neurogenic inflammation-related mediators may attenuate cough hypersensitivity in PIC models (Xu et al., 2024). However, the effective suppression of TRPV1/TRPA1 in animal models has not translated into spontaneous cough improvement with TRPV1 single-target antagonists in chronic cough patients; this clinical translation challenge is discussed in Section 5.2.

### 5.2 Dissociation between provoked cough efficacy and spontaneous cough inefficacy of TRPV1 antagonists

Clinical studies of TRPV1 antagonists have revealed a dissociation between *in vitro*/provoked efficacy and spontaneous cough inefficacy. SB-705498 elevated the capsaicin cough provocation threshold in chronic cough patients but failed to reduce 24-h objective cough frequency or significantly improve patient-reported outcomes (Khalid et al., 2014). The subsequent higher-potency TRPV1 antagonist XEN-D0501 demonstrated stronger suppressive effects in preclinical and capsaicin provocation tests but still did not reduce spontaneous awake cough frequency in patients with chronic refractory cough (Belvisi et al., 2017). These two studies collectively indicate that capsaicin-provoked cough primarily reflects TRPV1-related stimulus responses, whereas real-world chronic cough may be jointly maintained by the ATP-P2X3 axis, inflammatory mediator-sensitized TRP channels, and central sensitization among other factors.

Therefore, TRPV1 provocation tests cannot serve as a sufficient surrogate endpoint for predicting spontaneous cough efficacy. TRPV1 single-target blockade is insufficient to control chronic spontaneous cough, indicating that the contribution of TRP channels to cough hypersensitivity may vary according to patient endotype, trigger factors, and disease stage. Figure 2 and Table 1 present P2X3 antagonist and TRPV1 antagonist clinical trial results in parallel, providing a direct visualization of the differences between the two at the objective spontaneous cough endpoint. Notably, suppression of capsaicin-provoked cough confirms TRPV1 target engagement but does not establish TRPV1 as the dominant driver of spontaneous cough; the negative 24-h endpoint may reflect inadequate patient enrichment or insufficient coverage of non-TRPV1 pathways.

**TABLE 1:** Summary of key clinical trial evidence for P2X3 antagonists and TRPV1 antagonists.

Trial	Study design	Population	Intervention	Primary endpoint	Key results	Status	References
COUGH-1 (NCT03449134)	Global multicenter RDB placebo-controlled Phase III	RCC/UCC adults, cough ≥1 year, n = 730	Gefapixant 15/45 mg BID vs. placebo	Change in 24 h cough frequency at 12 weeks	45 mg group: 18.5% reduction (p < 0.05); 15 mg did not meet endpoint	Completed	(McGarvey et al., 2022)
COUGH-2 (NCT03449147)	Global multicenter RDB placebo-controlled Phase III	RCC/UCC adults, n = 1,314	Gefapixant 15/45 mg BID vs. placebo	Change in 24 h cough frequency at 24 weeks	45 mg group: 14.6% reduction (p < 0.05)	Completed	(McGarvey et al., 2022; Muccino et al., 2020)
Japan long-term safety (NCT03696108)	Open-label long-term extension	Japanese chronic cough patients, 52-week follow-up	Gefapixant 45 mg BID	Safety and tolerability	Taste AE 96% (45 mg group), 20% discontinuation; antitussive efficacy maintained	Completed	(Niimi et al., 2022)
RELIEF	RDB placebo-controlled Phase IIa	Chronic cough patients	Camlipixant multiple doses	Awake cough frequency	Significant reduction in awake cough frequency; low dysgeusia incidence	Completed	(Smith et al., 2025a)
SOOTHE	RDB placebo-controlled Phase IIb	Chronic cough patients	Camlipixant multiple doses	Cough frequency and dose optimization	200 mg group: ∼33% improvement in 24 h cough frequency; dysgeusia ∼10.6%, no discontinuation	Completed	(Smith et al., 2025b)
SB-705498 trial	RDB placebo-controlled Phase II	Chronic cough patients	SB-705498 vs. placebo	Capsaicin cough threshold +24 h spontaneous cough	Provocation test positive; no significant improvement in 24 h spontaneous cough	Terminated	(Khalid et al., 2014)
XEN-D0501 trial	RDB placebo-controlled proof-of-concept	Chronic cough patients	XEN-D0501 vs. placebo	Capsaicin cough +24 h spontaneous cough	Near-complete suppression in provocation test; no improvement in 24 h spontaneous cough	Terminated	(Belvisi et al., 2017)

### 5.3 P2X3 and TRP pathways are simultaneously activated following infection; single-target blockade is insufficient to cover all endotypes

The hypersensitive state in chronic cough may not result from a single channel abnormality, but rather from the simultaneous activation of multiple channels, inflammatory mediators, and neural circuits. TRP channels preferentially sense external physical and chemical stimuli as well as endogenous inflammatory metabolites, whereas the P2X3 receptor more directly responds to ATP released from epithelial damage and stress. During the post-infectious phase, incomplete epithelial repair, persistent inflammatory mediators, and altered sensory nerve channel expression may coexist, placing both pathway types in a state of heightened reactivity.

Cross-talk between P2X3 and TRP pathways warrants separate analysis. Experimental evidence demonstrates that TRPV1 activation can trigger the release of neuropeptides such as substance P (SP) and calcitonin gene-related peptide (CGRP) from sensory nerve terminals through Ca^2+^ influx; these neuropeptides can further damage airway epithelium and promote ATP release, thereby activating the P2X3 pathway and constituting a positive cascade from TRP to P2X3 (Xu et al., 2024; Al-Shamlan and El-Hashim, 2019). Conversely, P2X3 activation-induced depolarization can increase neuronal excitability, making TRPV1 and TRPA1 more readily reaching activation thresholds. This bidirectional potentiation implies that even if a patient initially presents with predominantly single-pathway activation, multi-pathway co-existing hypersensitivity may develop over time.

This synergistic network hypothesis also explains why P2X3 antagonists, although effective, achieve limited efficacy magnitude. For patients in whom the ATP-P2X3 axis predominates, P2X3 antagonists may be more effective; for patients with predominantly stimulus-induced cough, high TRPA1/TRPV1 sensitivity, or persistently elevated inflammatory mediators, P2X3 blockade alone may be insufficient. Future therapeutic strategies may need to select monotherapy, combination therapy, or microenvironmental modulation based on molecular endotype. Figure 3 illustrates how transcriptional and metabolic regulation jointly maintain P2X3 and TRP channel hyperexcitability.

**FIGURE 3:**
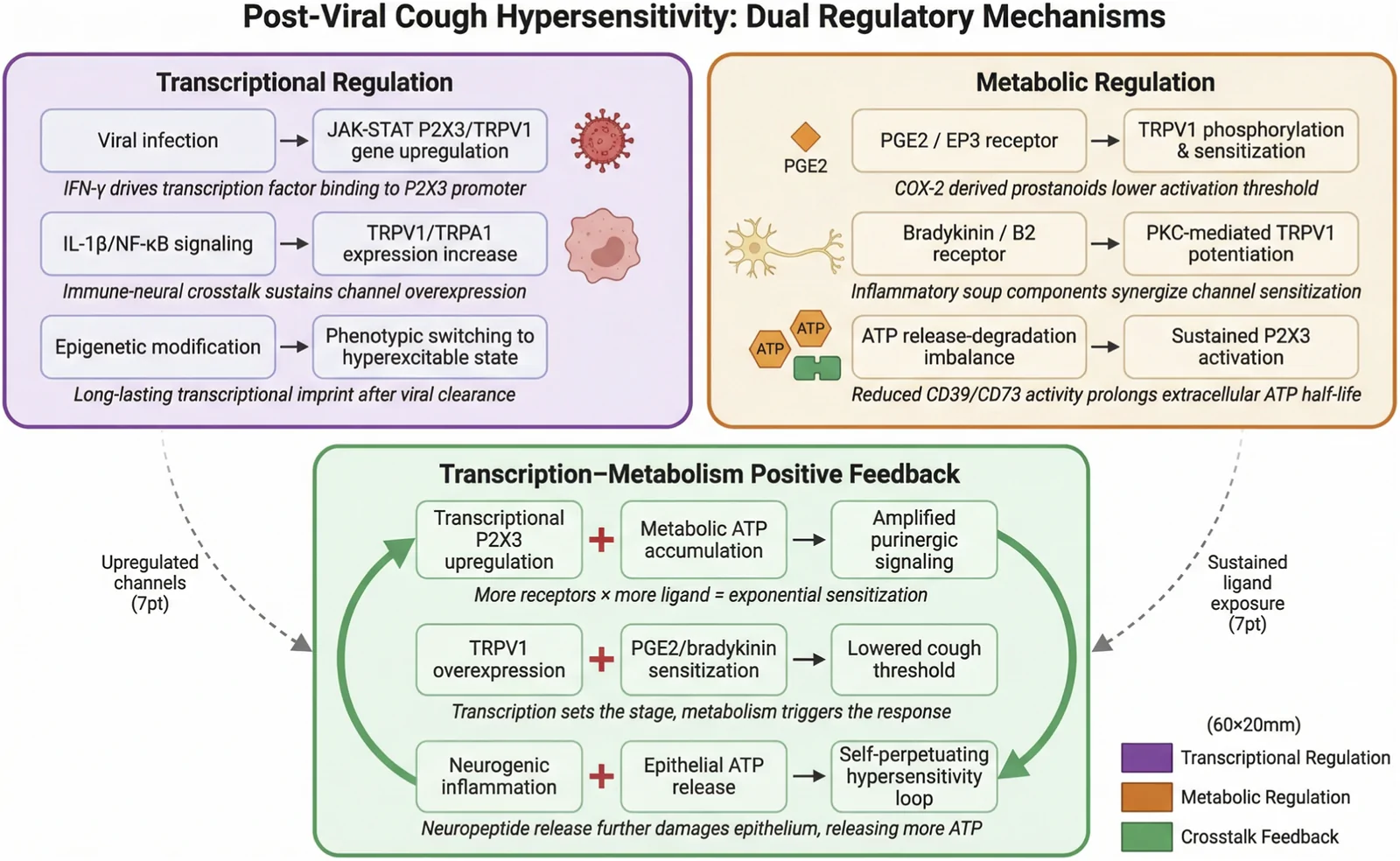
Interaction diagram of the dual dimensions of transcriptional and metabolic regulation.

## 6 Viral infection induces sustained sensory neuron hyperresponsiveness through transcriptional reprogramming

### 6.1 Rhinovirus and respiratory syncytial virus directly upregulate TRPV1, TRPA1, and ASIC3 expression

A key feature of PIC is that cough persists after resolution of acute infection. Its temporal persistence indicates that viral infection may not only induce transient inflammatory responses but may also alter intercellular signaling patterns among sensory neurons, airway epithelial cells, and immune cells, maintaining channel-related gene expression and cellular excitability at abnormal levels. Transcriptional regulation provides a mechanistic framework for explaining this sustained hypersensitivity.

Human epithelial and peripheral neuron model studies have provided model-based evidence for viral effects on sensory pathways. Rhinovirus infection can induce upregulation of antiviral response genes, sensory neuropathy-associated genes, and NMDA receptor activity-related genes in human epithelial and peripheral neuron models; IL-1β released from infected epithelial cells can also enhance neuronal TRPA1 responses (Dunne et al., 2025). Furthermore, respiratory syncytial virus and measles virus infection has been shown in experimental models to upregulate TRPV1, TRPA1, and ASIC3 expression in airway epithelial and neuronal cells, and the TRPV1 antagonist capsazepine can suppress this upregulation (Omar et al., 2017). PIC-related neuroimmune research has also indicated that infection-induced cytokines such as IFN-γ may participate in sensory nerve functional alterations (Kim and Song, 2022). These results support that viral infection can affect channel gene expression through two pathways: direct neuronal infection and indirect activation of epithelial-immune mediators. However, direct evidence for sustained upregulation of P2X3, TRPV1, and TRPA1 in sensory neurons from human PIC patients remains limited; the above models should currently be regarded as a hypothetical integrative framework.

### 6.2 Sensory neuron phenotypic transition from low-responsiveness to high-responsiveness can explain persistent post-infectious cough

Sensory neuron phenotypic plasticity refers to the alteration of receptor and ion channel expression profiles in neurons following inflammation, injury, or infection, thereby changing their response thresholds and response intensities to stimuli. If P2X3 receptor expression or membrane localization increases, lower concentrations of extracellular ATP can induce stronger depolarization and action potential discharge. If TRPV1 or TRPA1 expression is upregulated or sensitization is enhanced, patient cough responses to temperature changes, acidic environments, cold air, oxidative stress products, and irritant odors may also be enhanced.

This phenotypic transition has been relatively systematically described in the field of chronic pain. Inflammatory mediators such as nerve growth factor (NGF), bradykinin, and PGE2 can increase TRPV1 insertion into sensory nerve terminal membranes through TrkA receptor, PKA, and PKC signaling cascades, enabling neurons that originally responded only to temperatures above 42 °C to be activated at 37 °C (Chung et al., 2022; Mazzone and Undem, 2016). By analogy, similar mechanisms may contribute to low-threshold sensory nerve responsiveness after infection, but this remains a hypothesis requiring validation in PIC-specific human studies. NGF expression is elevated in airway inflammation and can increase TRPV1 and TRPA1 gene transcription through the PI3K/AKT pathway, while simultaneously promoting channel protein trafficking to the cell membrane through post-translational modifications (Yang et al., 2025). This means phenotypic plasticity involves two levels: long-term alterations at the gene transcription level that increase channel expression, and rapid sensitization at the post-translational level that lowers activation thresholds of already-expressed channels.

The persistent low-threshold cough observed in PIC patients therefore reflects not simply ongoing inflammation, but a sustained reduction in sensory afferent thresholds. In this framework, infection-associated cytokines first upregulate channel gene expression through JAK-STAT or NF-κB signaling (transcriptional level), while inflammatory metabolites concurrently sensitize existing channel proteins through kinase-dependent phosphorylation (post-translational level). The net result—increased channel density combined with lower activation thresholds—can maintain cough hypersensitivity well beyond the resolution of acute infection, although direct validation in human PIC sensory neurons remains lacking.

### 6.3 Sustained peripheral input can induce central sensitization; some residual cough originates from elevated central gain

Peripheral sensitization alone cannot fully explain all chronic cough phenomena. Sustained peripheral afferent input may induce functional alterations in brainstem cough centers and cortical regulatory regions, elevating central gain of the cough reflex and reducing patients' ability to suppress cough urges (Chung et al., 2022). This process shares similarities with the concept of central sensitization in chronic pain: sustained peripheral stimulation can lead to amplified central nervous system responses to subsequent inputs.

Functional brain imaging studies have indicated that capsaicin-evoked cough urge can activate regions of the human brain associated with sensation, emotion, and motor control (Mazzone et al., 2007). Neuroimaging studies of chronic cough patients have also demonstrated that cough hypersensitivity may be associated with central sensitization and dysfunctional inhibitory control (Ando et al., 2016). These findings indicate that PIC and chronic cough should not be viewed solely as peripheral channel disorders. For some patients, residual cough persisting after peripheral P2X3 or TRP channel blockade may be related to elevated excitability of central cough networks. Therefore, future stratified therapy should incorporate combined assessment of peripheral channel characteristics and central regulatory characteristics.

### 6.4 Direct transcriptomic data from sensory neurons of human PIC patients remain absent

Although virus-induced transcriptional alterations and sensory nerve plasticity have a plausible mechanistic basis, currently available direct evidence derives primarily from *in vitro* models, animal models, or indirect clinical observations (Dunne et al., 2025). Single-cell transcriptomic, spatial transcriptomic, or proteomic data from airway sensory neurons of human PIC patients are still lacking. Given the difficulty of obtaining tissues such as the nodose ganglion and jugular ganglion from patients, such research faces substantial technical and ethical constraints.

Future studies may consider utilizing induced pluripotent stem cell-derived sensory neurons, airway epithelial-neuron co-cultures, organoid models, peripheral blood inflammatory markers, and non-invasive neuroimaging markers to establish a translatable research framework. Simultaneously, data on symptoms, cough frequency, channel provocation tests, inflammatory mediators, and metabolomics should be collected in prospective cohorts before and after infection to identify molecular features associated with PIC onset, persistence, and treatment response. Only through patient-level validation of these features can the transcriptional regulation framework be translated into clinical subtyping tools.

## 7 The inflammatory microenvironment sustains channel hyperresponsiveness through post-translational modifications and ATP metabolic imbalance

### 7.1 Bradykinin sensitizes TRPV1 and TRPA1 channels through COX/12-LOX metabolites

Under infectious or inflammatory conditions, damaged epithelial cells and infiltrating immune cells release inflammatory mediators that can sensitize ion channels through two principal routes: direct phosphorylation of channel proteins by kinases (notably PKA and PKC), which lowers their activation thresholds, and transcriptional upregulation via intracellular signaling cascades such as MAPK/NF-κB and PI3K/AKT (Yang et al., 2025; Dunne et al., 2025; Su et al., 2025). Among these mediators, bradykinin and PGE2 have the most direct evidence linking inflammatory metabolites to TRP channel sensitization in the cough reflex.

Bradykinin research has provided direct evidence for inflammatory metabolite sensitization of TRP channels. Animal experiments demonstrated that bradykinin can activate COX and 12-LOX metabolic pathways through a B2 receptor-dependent mechanism, with metabolites further participating in TRPV1 and TRPA1 channel sensitization and enhancing the cough reflex. The sensitization mechanism of PGE2 has been studied in greater depth: PGE2 activates the Gs-AC-cAMP-PKA signaling axis through EP4 receptors; PKA can directly phosphorylate serine residues on the TRPV1 channel protein (Ser502, Ser800, and other sites), enabling TRPV1 activation below the normal temperature threshold. Additionally, the PKC pathway can also lower channel activation thresholds through phosphorylation of TRPV1 Ser502 and Ser800 sites (Al-Shamlan and El-Hashim, 2019). These post-translational modifications are reversible but can occur repeatedly in a sustained inflammatory microenvironment, maintaining channels in a sensitized state over prolonged periods.

PIC-related traditional Chinese medicine formula studies have also suggested in preclinical settings that the PI3K/AKT/TRP axis and MAPK/NF-κB inflammatory signaling pathways may participate in cough hypersensitivity maintenance (Yang et al., 2025; Su et al., 2025). These studies collectively indicate that channel function depends not only on expression levels but is also subject to sustained regulation by metabolites and signaling pathways within the inflammatory microenvironment (Table 2).

**TABLE 2:** Principal signaling pathways and molecular mechanisms associated with P2X3 and TRP channels in PIC.

Pathway	Key molecules	Upstream activators	Downstream effects	Pathological significance in PIC	Targeted agents	References
ATP-P2X3 epithelial-neural signaling axis	ATP, Pannexin channels, P2X3, P2X2/3	Epithelial damage (viral, mechanical, inflammatory)	P2X3 opening → Na^+^/Ca^2+^ influx → depolarization → cough reflex	Excessive ATP release + P2X3 upregulation → positive feedback loop	Gefapixant, Camlipixantetc.	(Bussey et al., 2026; Canning et al., 2026)
TRPV1 capsaicin receptor pathway	TRPV1, Ca^2+^, SP, CGRP	Capsaicin, high temperature, acid, PGE2, NGF	Ca^2+^ influx → C-fiber activation → cough + neurogenic inflammation	TRPV1 upregulation and sensitization; single blockade insufficient to suppress spontaneous cough	SB-705498, XEN-D0501	(Xu et al., 2024; Belvisi et al., 2017; Khalid et al., 2014)
TRPA1 chemosensory pathway	TRPA1, Ca^2+^, oxidative stress products	Cigarette smoke, formaldehyde, cold air, 4-HNE	Ca^2+^ influx → C-fiber activation → cough	TRPA1 upregulation, increased environmental chemical sensitivity; differential expression from TRPV1	Zhisou Powder, Kemin Capsule	(Xu et al., 2024; Long et al., 2019; Yang et al., 2025)
PI3K/AKT/mTOR pathway	PI3K, AKT, mTOR	NGF, EGF, IL-1β	Transcriptional upregulation of TRPV1/TRPA1; promotion of channel phosphorylation and membrane trafficking	Excessive PI3K/AKT activation → TRP channel transcriptional upregulation + functional sensitization	Kemin Capsule	(Yang et al., 2025)
MAPK/NF-κB inflammatory pathway	ERK1/2, JNK, p38, NF-κB	PAMPs, LPS, TNF-α, IL-1β, ATP	Pro-inflammatory gene transcription → IL-1β/TNF-α/IL-6/COX-2 release → neuronal sensitization	Inflammation–neural sensitization positive feedback	Keke Tablet	(Su et al., 2025; Zhang et al., 2024)
COX-2/12-LOX arachidonic acid metabolism	COX-2, 12-LOX, PGE2, 12-HETE	Cell damage → membrane phospholipids → arachidonic acid	PGE2 → EP receptors → indirect TRPV1 sensitization; 12-HETE → direct TRPV1 activation	Metabolic sensitizer accumulation → TRP channel hyperresponsiveness	No PIC-specific agents	(Al-Shamlan and El-Hashim, 2019)
JAK-STAT pathway	JAK1/2, STAT1/3, IFN-γR	IFN-γ, IL-6	Target gene transcription (including P2X3/TRPV1 promoter regions)	Viral immune response → neuronal transcriptional reprogramming	No PIC-specific agents	(Dunne et al., 2025; Kim and Song, 2022)
P2Y2-EGFR-ATP release positive feedback	P2Y2, EGFR, Pannexin-1	ATP → P2Y2 → EGFR transactivation → Pannexin-1 → more ATP release	Sustained elevation of ATP concentration → repeated P2X3 activation	Maintains excessive P2X3 activation after initial injury resolves	No specific targeting agents	(Zhang et al., 2024)
CD39/CD73 ATP degradation axis	CD39, CD73, adenosine	Extracellular ATP	ATP → ADP → AMP → adenosine → A2A/A2B receptors → anti-inflammatory	Reduced CD39/CD73 activity → ATP accumulation → sustained P2X3 activation	No PIC-specific agents	(Smith, 2024)

Pathways are arranged by functional hierarchy: rows 1–3 represent channel-level direct effector pathways; rows 4–7 represent intracellular signal transduction pathways; rows 8–9 represent metabolic regulatory pathways for ATP, release and degradation. Extensive cross-talk exists among these pathways; for example, NF-κB, is a key transcriptional regulator of COX-2, and the P2Y2-EGFR, pathway can directly activate MAPK/NF-κB, signaling.

### 7.2 Viral promotion of ATP release and inflammation-induced suppression of CD39/CD73 degradation lead to repeated P2X3 activation

ATP-P2X3 signal strength depends on extracellular ATP release quantity, diffusion range, receptor expression, and degradation rate. Under physiological conditions, extracellular ATP can be hydrolyzed by ectonucleotidases such as CD39 to ADP and AMP, then converted to adenosine by CD73, thereby terminating excessive purinergic signaling and participating in anti-inflammatory regulation. If infection or inflammation leads to increased ATP release or decreased degradation, ATP concentrations around nerve terminals may remain persistently elevated, causing repeated P2X3 receptor activation.

Virus-related epithelial stress can promote ATP release. The SARS-CoV-2 spike protein receptor-binding domain can induce ATP release from human bronchial epithelial cells and promote IL-6 and IL-8 release through downstream ATP/P2Y2-EGFR MAPK/NF-κB signaling (Zhang et al., 2024). Although this study focused on P2Y2 rather than P2X3, it indicates that viral components can amplify epithelial inflammatory signaling by promoting ATP release. On the other hand, sustained inflammation may suppress the expression or activity of ectonucleotidases such as CD39/CD73, reducing extracellular ATP clearance efficiency. When ATP release rate exceeds degradation rate, ATP concentrations around nerve terminals remain persistently elevated, and P2X3 receptors are repeatedly activated, thereby maintaining cough hypersensitivity.

### 7.3 Upstream inflammatory modulation and ATP metabolic intervention may supplement direct channel blockade strategies

From the metabolic regulation perspective, cough treatment should not be limited to direct ion channel blockade. If inflammatory mediators and ATP metabolic imbalance persist, single-receptor antagonism may only partially reduce afferent signaling without correcting the pro-sensitizing microenvironment. Therefore, upstream inflammatory signal modulation, ATP release inhibition, ectonucleotidase activity enhancement, and neurogenic inflammation control may all serve as supplementary strategies.

Currently, human evidence for these strategies in PIC remains insufficient. Inhibition of pathways such as MAPK/NF-κB or PI3K/AKT derives primarily from preclinical models (Yang et al., 2025; Su et al., 2025), and antitussive strategies enhancing CD39/CD73 activity remain at theoretical or early exploratory stages. Future research should determine the relative contributions of inflammatory metabolites, ATP metabolism, and channel expression in different patients and verify through randomized controlled trials whether microenvironmental modulation strategies can improve the efficacy of P2X3 or TRP-targeted therapy.

## 8 Therapeutic implications and future directions

### 8.1 PIC patients with different predominant pathways require distinct targeted strategies

Available evidence indicates that treatment of PIC and chronic cough needs to transition from symptom-based empirical therapy toward precision stratification based on molecular endotypes. For patients characterized primarily by increased ATP release, insufficient ATP degradation, or enhanced P2X3 expression and function, P2X3 antagonists may offer greater therapeutic specificity. For patients in whom exogenous stimulus-triggered cough predominates, with elevated TRPV1 or TRPA1 provocation sensitivity, TRP-related pathways may carry greater pathological weight; however, whether TRPV1 blockade alone is sufficient remains uncertain. For patients with persistently elevated inflammatory mediators or abnormal metabolic microenvironments, anti-inflammatory therapy, neurogenic inflammation modulation, or metabolic interventions may serve as necessary adjuncts. Although P2X3 and TRP channels are the focus of this review, cough persistence may also be influenced by non-ion-channel contributors, including airway remodeling, microbiome alterations, autonomic dysfunction, and behavioral cough components. These mechanisms may interact with sensory hypersensitivity and should be considered in future PIC phenotyping studies.

Achieving such stratified therapy requires clinically feasible and validated biomarkers (Table 3). The RCC/UCC field, which overlaps with but is not identical to PIC, currently lacks validated diagnostic or treatment-response predictive markers (Hassan et al., 2025). Near-term clinically feasible markers include objective cough frequency, validated cough-related quality-of-life scores, laryngeal sensory abnormality scores, and standardized capsaicin/ATP/AITC provocation sensitivity. In contrast, blood or airway inflammatory mediators, ATP metabolism-related enzymes, metabolomic profiles, and neuroimaging markers remain more exploratory because their cost, standardization, and accessibility may limit routine clinical use.

**TABLE 3:** Multi-dimensional comparison of four intervention categories.

Dimension	P2X3 antagonists (1st generation: Gefapixant)	P2X3 antagonists (2nd generation: Camlipixant et al.)	TRPV1 antagonists (SB-705498/XEN-D0501)	Traditional Chinese medicine formulas (KMC/ZSP/KKP)
Primary target	P2X3 + P2X2/3 (non-selective)	P2X3 (highly selective, sparing P2X2/3)	TRPV1	Multi-target (PI3K/AKT/TRP; MAPK/NF-κB)
Level of action	Peripheral sensory nerve terminals (blocking ATP-P2X3 signaling)	Same as above, with higher selectivity	Peripheral C-fibers (blocking chemical/thermal sensing)	Multi-level (transcription + microenvironment + channel function)
Highest evidence level	Phase III RCT (n > 2000)	Phase IIb RCT	Phase II RCT	Preclinical animal models
Spontaneous cough efficacy	Positive (24 h cough frequency reduced 14.6%–18.5%)	Positive (significant reduction in awake cough frequency)	Negative (no reduction in 24 h spontaneous cough)	Positive in animal models; no human data
Provoked cough efficacy	Not a primary endpoint	Not a primary endpoint	Strongly positive (near-complete capsaicin suppression)	Not evaluated
Key safety concerns	Dysgeusia >50% (P2X2/3 off-target)	Dysgeusia substantially reduced	No significant safety signals	Lacking systematic toxicology data
Transcriptional regulation effects	No direct effect on channel transcription	Same as above	No direct effect	May indirectly modulate via PI3K/AKT or MAPK/NF-κB
Metabolic regulation effects	Blocks ATP signaling but does not alter release/degradation balance	Same as above	Blocks TRPV1 signaling but does not affect sensitizer levels	May reduce pro-sensitizing metabolites (PGE2, cytokines)
PIC-specific evidence	Derived from RCC/UCC; no PIC-specific RCT	Same as above	Same as above	Some studies employed PIC animal models

### 8.2 Implications for P2X3 antagonist development

The clinical value and limitations of gefapixant have together driven the development of second-generation highly selective P2X3 antagonists. The design rationale is to maintain inhibitory activity against P2X3 homotrimers while minimizing activity against P2X2/3 heterotrimers implicated in taste signaling, thereby reducing dysgeusia (Friedri et al., 2022; Francke et al., 2025). However, higher P2×3 selectivity does not necessarily translate into stronger objective antitussive efficacy, as illustrated by the failure of sivopixant to meet its phase IIb primary endpoint (McGarvey et al., 2023). These findings suggest that future P2X3 antagonist development should balance receptor selectivity, tolerability, target engagement, and patient selection rather than relying on selectivity alone (Table 4).

**TABLE 4:** Comprehensive summary of candidate drugs and intervention strategies.

Drug/Strategy	Category	Target	Development stage	Key endpoint data	Safety	References
Gefapixant (MK-7264)	P2X3/P2X2/3 antagonist (1st generation)	P2X3, P2X2/3	PMDA approved (Japan); FDA CRL	Phase III: reduced 24 h cough frequency	Dysgeusia >50%; Japanese long-term study: taste AE 96%, 20% discontinuation	(McGarvey et al., 2022; Muccino et al., 2020; Niimi et al., 2022)
Camlipixant (BLU-5937)	Selective P2X3 antagonist (2nd generation)	P2X3	Phase IIb (completed)	Reduced awake/24 h cough frequency	Dysgeusia ∼10.6%; no discontinuation due to dysgeusia	(Smith et al., 2025a; Smith et al., 2025b)
Sivopixant (S-600918)	Selective P2X3 antagonist (2nd generation)	P2X3	Phase IIb (primary endpoint not met)	No significant 24 h cough-frequency benefit; symptom benefit observed	Lower dysgeusia incidence	(McGarvey et al., 2023)
Filapixant (BAY1902607)	Selective P2X3 antagonist (2nd generation)	P2X3	Phase II (completed)	Reduced cough frequency/severity; improved quality of life	Dysgeusia lower than gefapixant	(Francke et al., 2025)
Representative TRPV1 antagonists (SB-705498; XEN-D0501)	TRPV1 antagonists	TRPV1	Phase II (terminated)	Suppressed provoked cough; no spontaneous-cough benefit	Well tolerated; limited by insufficient efficacy	(Belvisi et al., 2017; Khalid et al., 2014)
Representative traditional Chinese medicine formulas (KMC; ZSP; KKP)	Multi-target traditional Chinese medicine interventions	PI3K/AKT/TRPV1/TRPA1; MAPK/NF-kB	Preclinical	Improved cough-related phenotypes in PIC models	Historical/traditional safety support; no high-quality human RCT data	(Xu et al., 2024; Yang et al., 2025; Su et al., 2025)

### 8.3 Existing RCC/UCC trials cannot be directly extrapolated to PIC; PIC-specific randomized controlled trials are needed

Current large-scale clinical trials of P2X3 and TRP antagonists have primarily enrolled RCC/UCC patients (McGarvey et al., 2022; Muccino et al., 2020). Cohort observations confirm this distinction in disease trajectory: in a nationwide prospective cohort of 1,650 patients with post-COVID-19 post-infectious cough, cough resolved in 90.8% within 32 weeks and only 6.4% persisted beyond 1 year, whereas the mean cough history of patients enrolled in RCC/UCC trials exceeded 10 years (He et al., 2026; McGarvey et al., 2022; Muccino et al., 2020). These populations have prolonged disease duration and complex etiologies; even if PIC patients are included, they are typically not analyzed as an independent subgroup. Therefore, direct extrapolation of overall RCC/UCC study results to PIC involves uncertainty. PIC has a defined infectious prodrome and may differ from non-infection-related chronic cough in terms of virus-induced epithelial injury, neuroimmune responses, channel expression alterations, and ATP metabolic imbalance.

Future clinical research should consider two study designs (Figure 4). First, pre-specified PIC subgroup analyses within large chronic cough trials should document infection type, time from infection to cough persistence, inflammatory biomarkers, provocation test results, drug exposure, pharmacodynamic target-engagement readouts, and treatment response to characterize the response profile of PIC patients to P2X3 or other targeted agents. Second, directly conducted PIC-specific randomized controlled trials with defined disease duration and infection evidence as inclusion criteria, employing objective cough frequency, cough-related quality of life, laryngeal sensory abnormality scores, and biomarkers as co-primary or composite endpoints. Table 1 lists the available major clinical trial evidence and demonstrates that PIC-specific evidence remains notably insufficient.

**FIGURE 4:**
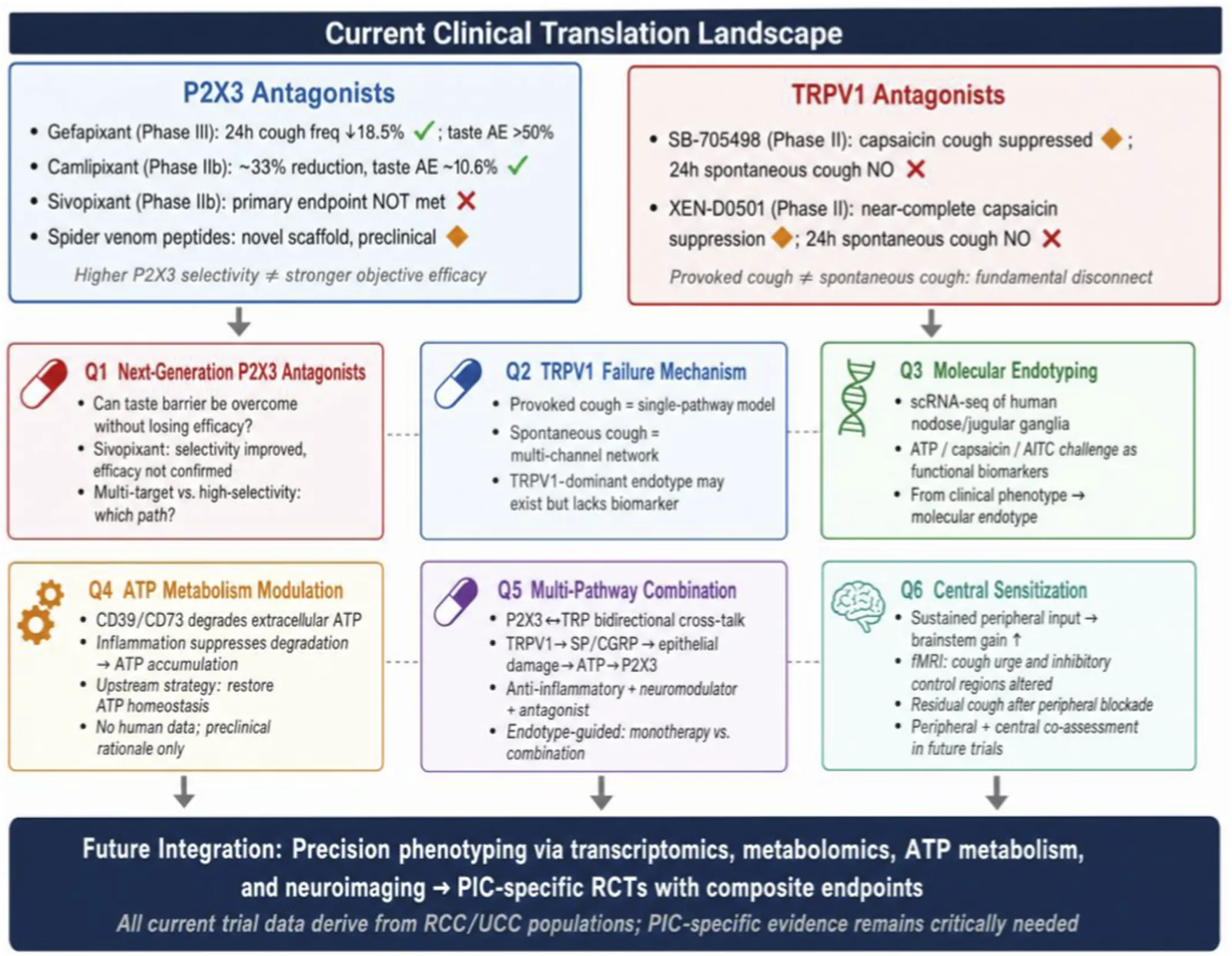
Conceptual framework of principal challenges and future directions for P2X3 and TRP channel-targeted therapy in post-infectious cough.

## 9 Conclusion

This review integrated evidence for P2X3 and TRP channel hyperexcitability in PIC from the dimensions of transcriptional regulation and metabolic regulation, and used this framework to explain the potential mechanisms underlying the clinical efficacy differences between these two classes of antagonists. The principal conclusions are as follows.

First, the pathological basis of PIC is a reduction in airway sensory nerve excitation threshold and enhancement of responsiveness. Beyond cough, PIC patients frequently present with laryngeal sensory abnormalities and cough triggered by low-intensity stimuli, indicating that the pathological process involves multiple levels including upper airway sensory afferents, the ATP-P2X3 signaling axis, TRP channel sensitization, and central regulatory abnormalities. As one etiology of subacute cough (i.e., cough of 3–8 weeks' duration (Irwin et al., 2018)), PIC requires comprehensive evaluation incorporating disease duration, comorbidities, and sensory nerve hyperresponsiveness characteristics, rather than broad diagnosis based solely on an infectious prodrome.

Second, the clinical efficacy difference between P2X3 antagonists and TRPV1 antagonists does not reflect that a single pathway independently determines the disease, but more likely reflects differences in the relative contributions of channel expression, inflammatory microenvironment, ATP metabolism, and central regulation across individual patients. The reduction in objective cough frequency by P×23 antagonists in RCC/UCC supports the ATP-P2X3 axis as a targetable pathway in chronic cough; the effectiveness of TRPV1 antagonists in capsaicin provocation tests coupled with their failure to reduce spontaneous cough indicates that capsaicin provocation models cannot adequately represent the multifactorial pathology of real-world chronic cough. Despite possessing higher P2×3 selectivity, sivopixant failed to meet its phase IIb primary endpoint, further indicating that merely increasing receptor selectivity does not equate to stronger objective antitussive efficacy.

Third, the sustained hyperexcitability of P2X3 and TRP channels in PIC may be jointly maintained by two converging mechanisms: at the transcriptional level, viral infection and infection-induced cytokines (e.g., IFN-γ) can upregulate channel gene expression through JAK-STAT and NF-κB signaling; at the post-translational level, inflammatory mediators such as PGE2 and bradykinin can sensitize existing channel proteins through kinase-dependent phosphorylation, while impaired CD39/CD73-mediated ATP clearance sustains repeated P2X3 activation. However, this integrative framework currently rests on *in vitro* and animal model evidence; direct demonstration of persistent channel upregulation in human PIC sensory neurons is still lacking.

Fourth, future research should prioritize the following directions: (1) establishment of a PIC molecular endotype classification system integrating objective cough frequency, channel provocation sensitivity, inflammatory mediators, ATP metabolism enzymes, metabolomic profiles, and neuroimaging markers; (2) optimization of the safety of highly selective P2X3 antagonists and exploration of comprehensive therapeutic strategies combining peripheral channel blockade, inflammatory microenvironment modulation, and central sensitization intervention; (3) conduct of PIC-specific randomized controlled trials with defined disease duration and infection evidence, employing objective cough frequency, cough-related quality of life, laryngeal sensory abnormality scores, and biomarkers as composite endpoints.

This review has several limitations. First, as a descriptive rather than systematic review, literature selection may be subject to certain bias. Second, direct evidence for virus-induced transcriptional alterations in sensory neurons derives primarily from *in vitro* models and animal studies, with limited validation data at the neural tissue level in human PIC patients. Third, the dual-dimensional model of transcriptional and metabolic regulation represents a mechanistic integration based on available evidence and requires validation through prospective cohorts, PIC-specific randomized controlled trials, and multi-omics studies.
